# Reinforcement Positioning in Custom‐Made Mouthguards for Maxillofacial Trauma Protection: A Combined In Vitro and In Silico Analyses

**DOI:** 10.1111/edt.13060

**Published:** 2025-03-27

**Authors:** Talita Suelen de Queiroz, João Paulo Mendes Tribst, Larissa Haddad e Borro, Guilherme da Rocha Scalzer Lopes, Alexandre Luiz Souto Borges, Tarcisio Jose de Arruda Paes Junior

**Affiliations:** ^1^ Department of Dental Materials and Prosthodontics São Paulo State University (UNESP) São José Dos Campos São Paulo Brazil; ^2^ Department of Reconstructive Oral Care, Academic Centre for Dentistry Amsterdam (ACTA) University of Amsterdam and Vrije Universiteit Amsterdam Amsterdam the Netherlands

**Keywords:** biomechanical response, dental trauma, finite element analysis, mouthguard, reinforcement mesh

## Abstract

**Background/Aim:**

This study evaluated the dentoalveolar responses of central incisors to anterior maxillary trauma in vitro and in silico using mouthguards (MGs) reinforced with polyamide mesh at three distinct positions.

**Material and Methods:**

Forty 4‐mm thick MGs were categorized based on mesh location: Group MG1 + 3 (reinforcement 1 mm from the vestibular limit), Group MG2 + 2 (2 mm), Group MG3 + 1 (3 mm), and a control group without reinforcement. A 3D‐printed skull model (Spin Red Resin, Quanton 3D) simulated the dentoalveolar complex, with Resilab Clear resin (Wilcos) for teeth and addition‐cured silicone for the periodontal ligament. This setup was connected to a custom impact device to ensure forces remained within the materials' elastic limits. Microstrains were measured using four strain gauges placed on the vestibular surfaces of the central incisors and the alveolar process of the maxilla. The impact was applied at Ep = 0.5496 J, parallel to the ground, using a 35‐mm diameter steel sphere. For the in silico test, the setup was modeled in CAD software (Rhinoceros 7.0) and analyzed in CAE software (Ansys 2021 R1) through explicit dynamic simulation. All materials were assumed homogeneous, isotropic and linearly elastic. A 1 m/s impact was simulated using a 7.8 g/cm^3^ steel sphere. Physical contact conditions were defined as frictional and glued, with tetrahedral mesh elements applied after a 10% convergence test to ensure accuracy.

**Results:**

The maximum principal strains and stresses in teeth and maxilla were presented through colorimetric graphs. Statistical analysis (Shapiro–Wilk, Kruskal–Wallis, and Dunn's tests, 5% significance) revealed significant differences for the non‐reinforced group (*p* = 6.8 × 10^−5^) but none between impact zones (*p* = 0.879), confirming uniform stress distribution.

**Conclusions:**

Reinforcement systems significantly improved impact absorption in oral tissues, enhancing protection. However, the reinforcement location did not significantly affect absorption. Finite element analysis validated the in vitro results supporting both theoretical and practical models for further study and future improvements.

## 1 Introduction

Sports activities provide numerous benefits to both amateur and elite athletes; nevertheless, they also demand attention to injury prevention, particularly for orofacial trauma, which is common in high‐velocity, high‐altitude, and contact sports. In response, the American Dental Association (ADA) recommends the use of mouthguard devices [1, 2] to prevent not only tooth fractures and displacements but also mandibular and maxillary fractures, as well as soft tissue injuries, thereby reducing laceration occurrences [3, 4, 5, 6]. However, even with mouthguards (MGs) that meet ideal specifications, orofacial trauma remains a concern during sports impacts [1, 2].

The material used in MG fabrication can significantly influence its protective efficacy [7]. Ethylene‐vinyl acetate (EVA) is widely used and recommended due to its favorable mechanical properties, enabling impact absorption [1, 2, 8]. Additionally, EVA is easy to handle and allows the inclusion of reinforcement layers within the MG [1, 2]. For adequate impact absorption, EVA‐based MGs are typically fabricated with a thickness of 3–4 mm [8].

Custom‐made MGs may offer the advantage of minimal interference during speech, breathing and dry mouth sensation, but they can also provide superior adaptation to the oral cavity and reduce nausea when compared to the others available on the market, thereby aligning closely with ideal parameters for their use in contact sports [9]. Although MGs are essential for reducing or preventing orofacial injuries during sports activities, especially to the maxillary central incisors, they do not eliminate trauma in all cases; even using this dispositive during impacts, the stresses affecting the orofacial structures are relevant [1, 2]. Consequently, studies have been conducted to enhance MG efficacy through reinforcement methods, including laminated layers, air chambers [10], sorbothane inserts [11], acrylic resin [12], silica mesh [1], titanium [8], sponges, and fiberglass [13]. Nevertheless, the literature remains inconclusive regarding the most effective MG reinforcement method [1, 8].

Silica‐modified nylon fibers can be an option for reinforcing MGs during fabrication, [1] and the efficacy of these fiber reinforcements depends on their structural design, orientation, applied force and bond strength with the underlying material to enhance load distribution [14]. Additionally, one of the most effective methods to improve the mechanical properties of polymers is through the incorporation of silica nanoparticles, which enhance both mechanical and thermal characteristics [15]. In line with these advancements, researchers at the Institute of Science and Technology of São José dos Campos/UNESP‐SP developed a patented 0.5% silica‐reinforced nylon mesh (patent number BR1020120281198) to optimize load distribution [16]. This reinforcement structure, previously integrated into temporary fixed prostheses made of acrylic and bis‐acrylic resins [14] and full‐arch mucosa‐supported and implant‐supported prostheses [16, 17], has shown increased structural resistance. However, its application in MGs and the optimal placement of this reinforcement within the device remain subjects of ongoing research [1].

Finite element analysis (FEA), an in silico methodology, has proven to be a reliable approach for assessing stress and strain under load applications in complex structures through numerical modeling. This technique supports the study of impact mechanics by identifying regions susceptible to structural failure during trauma and enables detailed interpretation of trauma‐induced responses in biological tissues in a controlled and individualized behavior—a critical advantage given the challenges of replicating maxillofacial trauma in situ [18]. Nevertheless, the validation of mathematical models requires complementary experimental testing. A strain gauge, which quantifies local strain by measuring stress with sensitive analog sensors, shows itself to be an available test for evaluating impact absorption [19].

Given the need to develop MGs capable of suppressing stress during trauma, it is crucial to investigate the potential of reinforcing MGs in various configurations by combining in silico and in vitro methodologies to achieve the most accurate results possible [20]. Therefore, this study aimed to evaluate the dentoalveolar responses to anterior maxillary trauma, using 4 mm thickness MGs reinforced with a polyamide mesh in three different locations: 1 mm from the MG's vestibular limit (MG1 + 3), 2 mm (MG2 + 2), and 3 mm (MG3 + 1). The null hypotheses of this study were: (1) MGs with reinforcement systems do not influence impact absorption for oral tissues, and (2) modifying the reinforcement location within MGs does not influence impact absorption for oral tissues.

## 2 Materials and Methods

### 2.1 Finite Element Analysis

This study was conducted using three‐dimensional (3D) finite element analysis (FEA) with computer‐aided engineering software (ANSYS 2021‐R1; ANSYS Inc., Houston, TX, USA) to perform an explicit dynamic analysis of the affected dentoalveolar structures.

**FIGURE 1:**
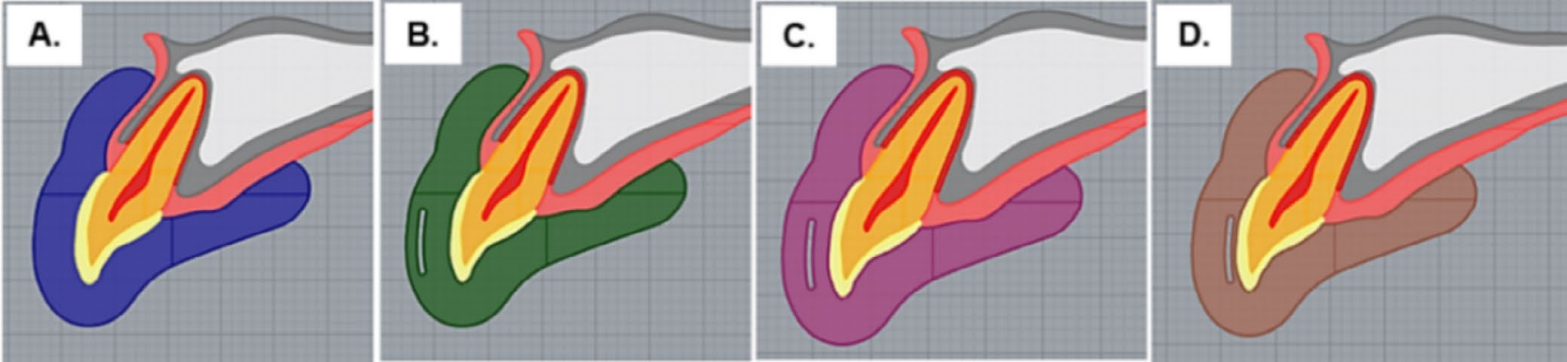
Different mesh placements within the MG in the vestibular direction according to the experimental groups. (A) 4 mm EVA MG without reinforcement (control); (B) 4 mm EVA MG with reinforcement 1 mm from the vestibular surface; (C) 4 mm EVA MG with reinforcement 2 mm from the vestibular surface; (D) 4 mm EVA MG with reinforcement 3 mm from the vestibular surface.

**FIGURE 2:**
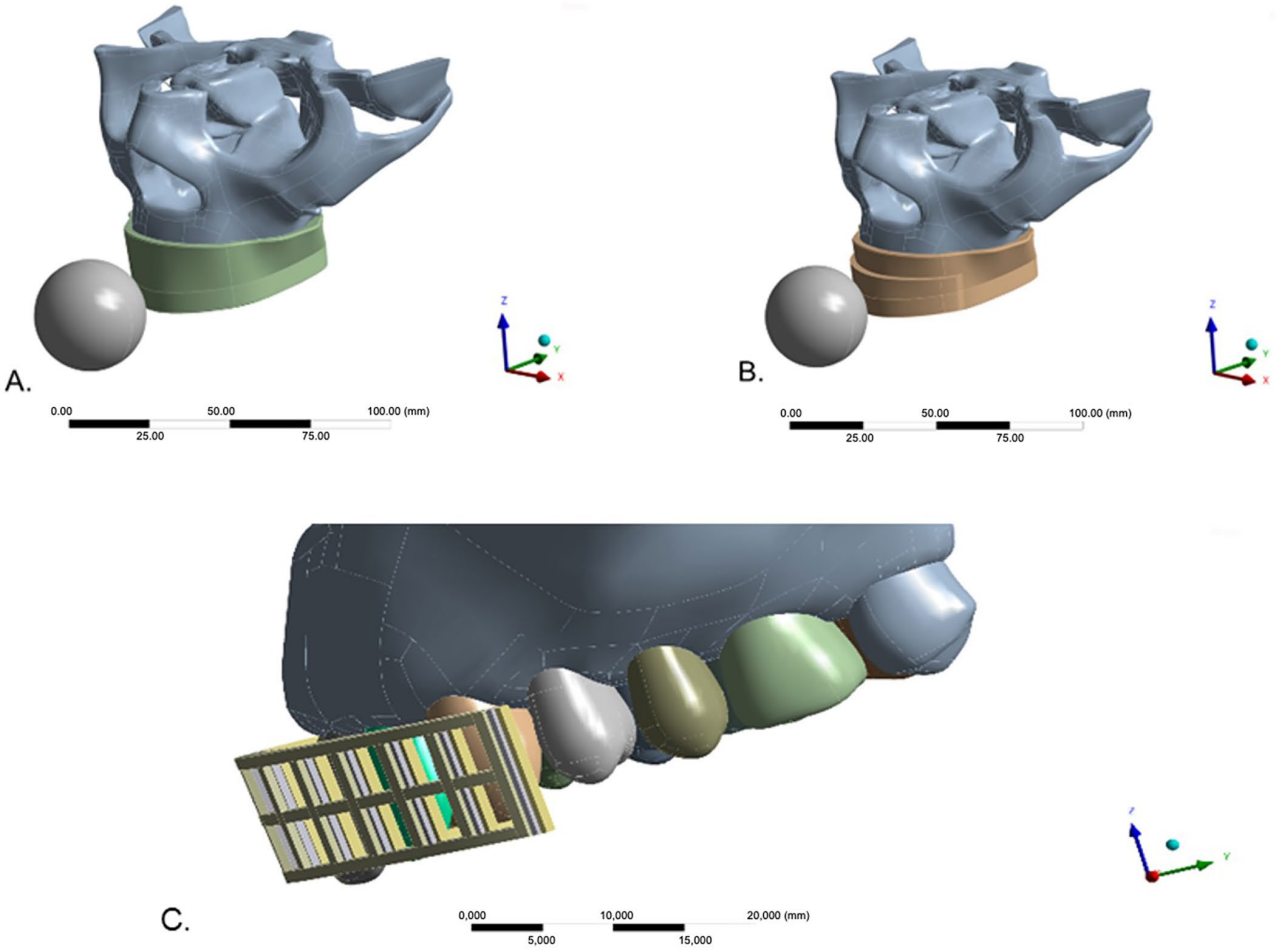
3D skull model illustrating conventional and reinforced MGs. (A) Skull model with non‐reinforced EVA MG; (B) Skull model with EVA MG reinforced with nylon mesh; (C) Skull model representing three reinforcement placements in relation to the vestibular limit of the teeth.

For modeling, a 3D mathematical skull model was imported via computer‐aided design (CAD) software (Rhinoceros version 7.0; McNeel North America, Seattle, WA, USA), as shown in Figure 1. A custom‐made 4 mm thick MG model was created, then replicated three times to include an experimental silica‐reinforced nylon mesh with a thickness of 0.6 mm, spaced at 3 mm and placed at three different positions: 1, 2, and 3 mm from the vestibular limit of the MG in the horizontal direction (Figures 1 and 2). Vertically, the reinforcement extended over the clinical crowns from canine to canine (teeth 13–23).

**FIGURE 3:**
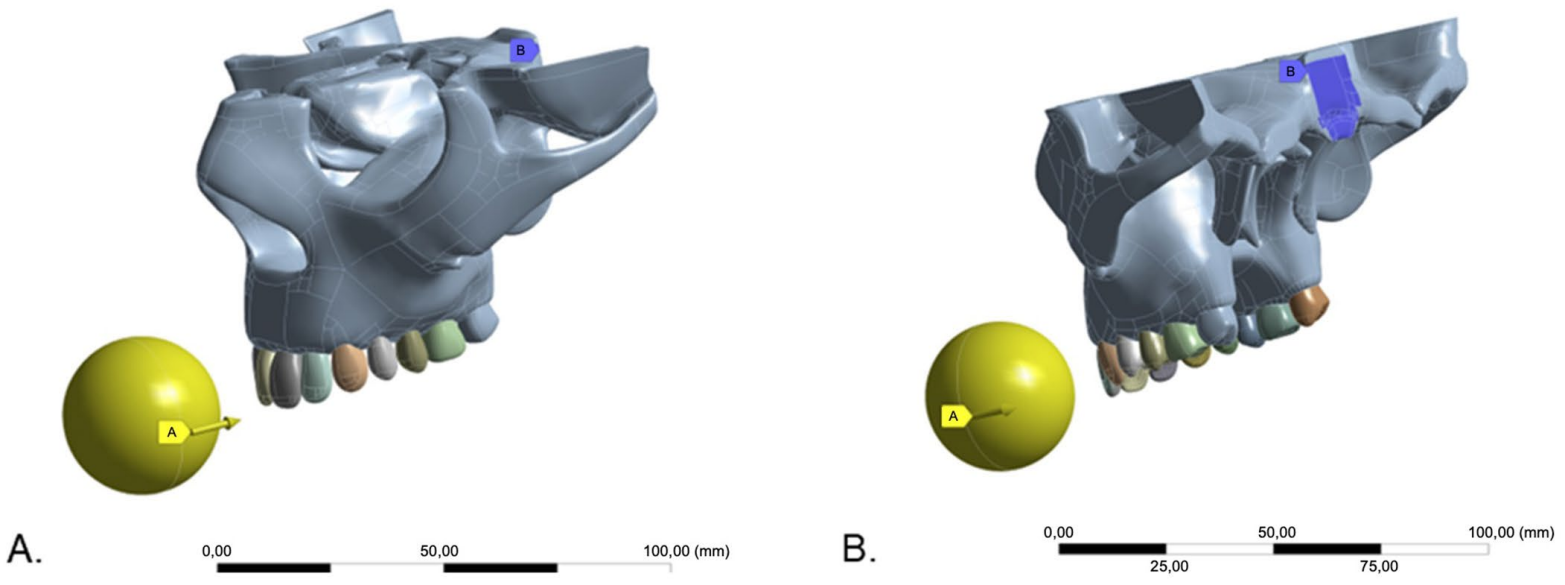
Illustration of the load application area using a steel sphere and constriction system in the posterior frontal section. (A) Yellow sphere with arrow indicating load application; (B) Blue arrow indicating the constriction system region simulating spinal column insertion.

All models were constrained at the posterior section of the frontal cut, simulating the connection to the spinal column (Figure 3). The load was applied to the upper central incisors using a 35 mm steel sphere (Figure 3), simulating an impact at 1.6 m/s and analyzed over 0.05 s, with an initial force of 0 N, increasing to a final force of 27.5 N to represent the collision condition for a protected tooth as per the in vitro study.

Elements were defined as tetrahedral 10 nodes (TET 10), with models averaging 65.654 elements and 17.350 nodes, determined by a 10% mesh convergence test. An ideal contact interface was established between all elements, defined as frictional between the MG and the steel ball, and bonded for the other contacts, simulating a condition where the athlete would not lose the MG upon impact.

The mechanical properties (elastic modulus and Poisson's ratio) of the materials used in the study are listed in Table 1, based on data from the literature. All materials in the system were considered homogeneous, isotropic, and linearly elastic.

**TABLE 1:** Mechanical properties of the materials.

Material	Elastic modulus (MPa)	Poisson's ratio	Density (g/cm^3^)
Resilab resin	4.000 [21]	0.8 [21]	0.7 [21]
Periodontal ligament	50 [22]	0.45 [22]	0.95 [22]
Spin red resin	3.420	1.078	0.7
Soft tissue	1.8 [23]	0.30 [24]	0.95 [24]
EVA	18.000 [23]	0.30 [23]	0.95 [25]
Steel	200.000 [23]	0.30 [23]	7.8 [25]
Nylon fiber	1900 [26]	0.17 [23]	1.2295 (Own measurement)

Stress (MPa) and strain (με) distribution results are presented using color scales, where impact absorption capacity was defined as a percentage of the peak stress compared between reinforced and non‐reinforced MG models over the teeth from 13 to 23, with no positional or physiological alterations. Maximum principal stress was a common failure criterion used to identify tensile stress regions in dental structures, while system displacement was used to assess the effect of silica reinforcement on MG flexion.

### 2.2 Validation of the Computational Model

#### 2.2.1 Specimen Preparation and 3D Printing of the Model in Resin

In order to validate the computational results, an in vitro study was conducted under similar conditions. For the 3D printing of the skull model simulating the mechanical properties of bone tissue, it was essential to confirm compatibility with the mechanical properties of polyurethane (F160, Axson Technologies, Saint‐Ouen‐I'Aumône, France), a material validated and frequently used to represent bone structures in vitro studies due to its elasticity and stiffness [27]. Three resins were selected—Spin Red, IRON, and RPG, all from Quanton 3D. Standardized samples were produced in dimensions of 60 × 10 × 5 mm and polished on a polishing machine (Skill‐Tec PSK‐2 V—Erios) with sandpapers of 600, 800, and 1200 grit to eliminate surface irregularities.

Mechanical properties analyses were performed using the ATCP Sonelastic device and interpreted in Sonelastic 2.8 software, based on the Impulse Excitation Technique (ASTM‐E1876), obtaining data on elastic modulus, torsional modulus, and Poisson's ratio, allowing comparison with validated polyurethane material. This non‐destructive mechanical characterization test consists of striking the samples with a controlled mechanical impulse while recording the vibrational response. The resonance frequencies obtained were used to calculate the properties previously mentioned. Each of the three samples selected was subjected to five tests, and the results were averaged as shown in Table 2 in order to assess the mechanical behavior of the resins stiffness and elasticity. These parameters are fundamental for selecting the most suitable resin for the in vitro test and integrating data for finite element analysis, ensuring accurate computational analysis.

**TABLE 2:** Mechanical properties of the analyzed resins.

Property	Spin red resin—Quanton 3D	RPG resin—Quanton 3D	Iron resin—Quanton 3D	Polyurethane (F160, Axson Technologies)
Elastic modulus (GPa)	3.4 ± 0.17	1.6 ± 0.11	2.1 ± 0.04	3.6 [28]
Torsional modulus (GPa)	1.1 ± 0.06	0.5 ± 0.04	0.7 ± 0.02	1.4 [28]
Poisson's ratio	0.6 ± 0.07	0.5 ± 0.04	0.6 ± 0.06	0.3 [29]

**FIGURE 4:**
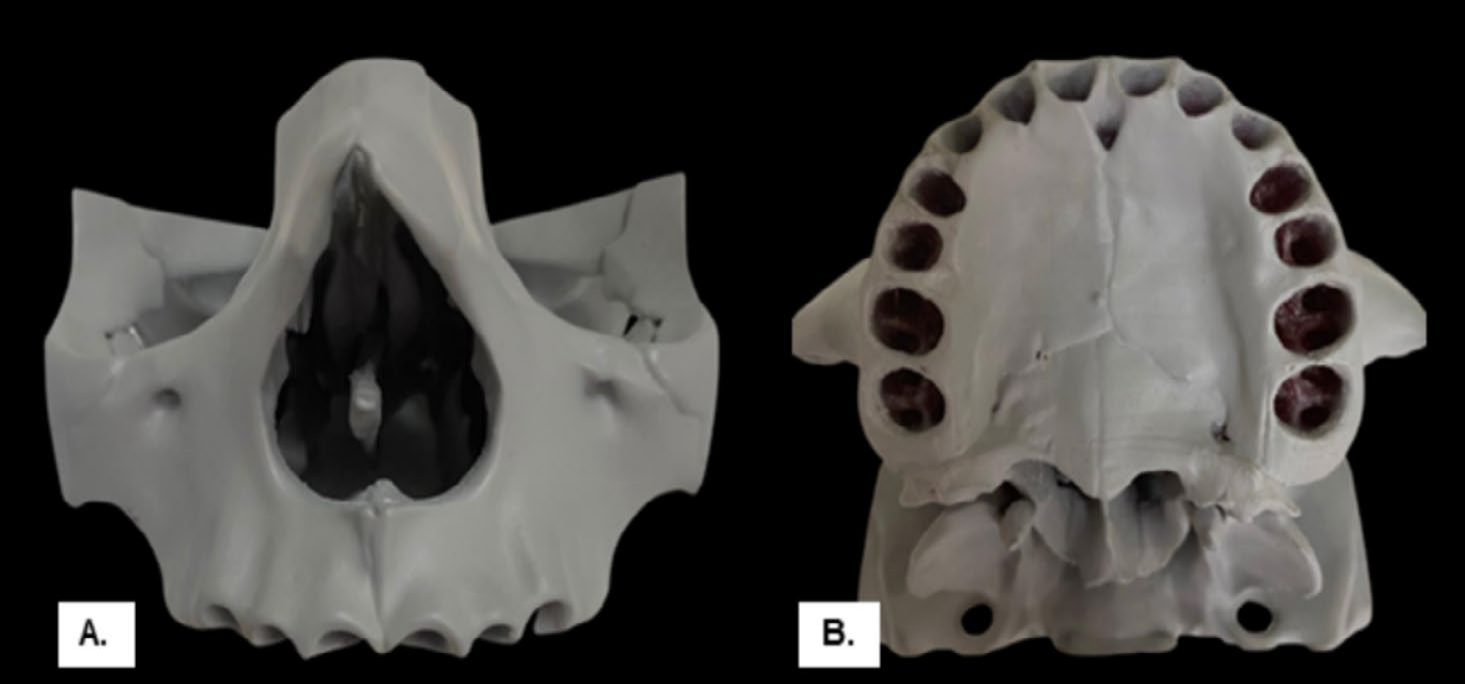
Skull 3D printed in spin red resin—Quanton 3D. (A) Frontal view. (B) Occlusal view.

Based on the data, Spin Red Resin—Quanton 3D showed polyurethane‐compatible values, which is the material currently validated for simulating bone structures in in vitro studies (Table 2). After determining the most appropriate resin, a 3D printer (GTMax3D Core A3) was used to print a maxilla model in STL format without teeth, which were subsequently printed separately using a W3D Print—Wilcos 3D printer and Resilab Clear resin—Wilcos. These models were designed in CAD software (Rhinoceros version 7.0; McNeel North America, Seattle, WA, USA) with dimensions of 5.6 cm in height, 8.6 cm in width from a frontal view, and 8.9 cm in the anteroposterior direction (Figure 4).

To simulate the periodontal ligament and securely seat each tooth in the printed model, an addition silicon material (Variotime Light Flow—Kulzer) was applied to the alveolar sockets of the skull model printed in Spin Red Resin.

Prior to molding the skull for MG fabrication, necessary precautions were taken regarding the future positions of strain gauges to avoid damaging them during impact tests.

#### 2.2.2 Mouthguard Fabrication

The upper dental arch of the 3D printed maxilla model was molded with alginate (Jeltrate Plus—Dentsply Sirona), following the manufacturer's instructions, to create a type IV dental plaster model, which was used in subsequent laboratory steps for MG fabrication.

Ten samples were created for each of the four MG groups (*n* = 10) and the MG fabrication involved pressing circular EVA sheets (Bio‐Art Equipamentos Odontológicos Ltda.) of original size 134 × 134 mm and 4 mm thickness on the plaster model using a vacuum forming machine (Plastvac—P7, Bioart) to produce the control group of non‐reinforced MGs.

**FIGURE 5:**
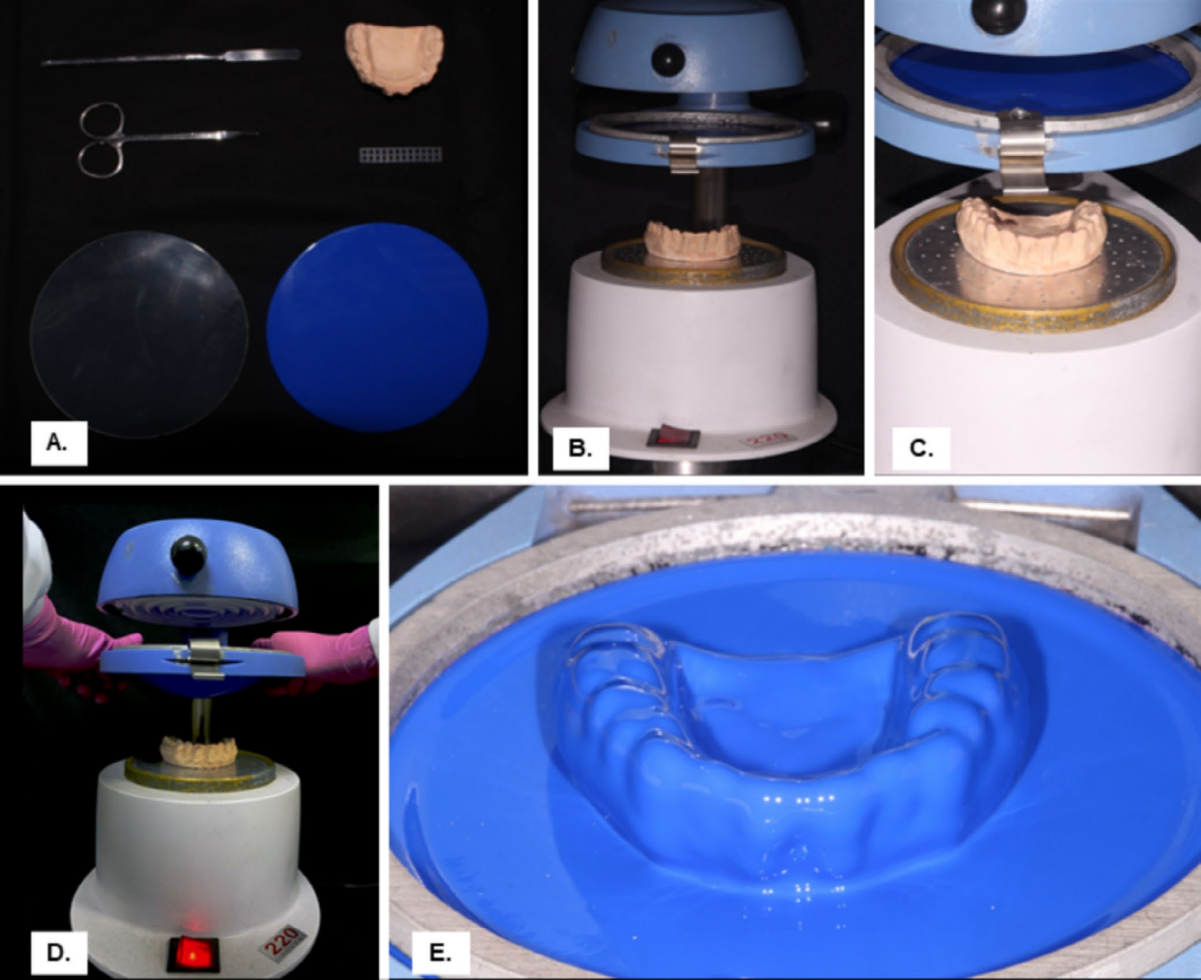
MG fabrication with silica‐reinforced nylon mesh. (A) Setup with necessary tools for reinforced MG fabrication, exemplified here with a 3 mm sheet (blue), reinforcement (silica‐incorporated nylon mesh), and 1 mm sheet (transparent); (B) Plaster model in the vacuum‐forming machine (Plastvac – P7, Bioart) with a 3 mm soft EVA sheet (blue); (C) Close‐up of initial vacuum forming of the 3 mm EVA sheet; (D) Completed vacuum forming; (E) Final pressing.

**FIGURE 6:**
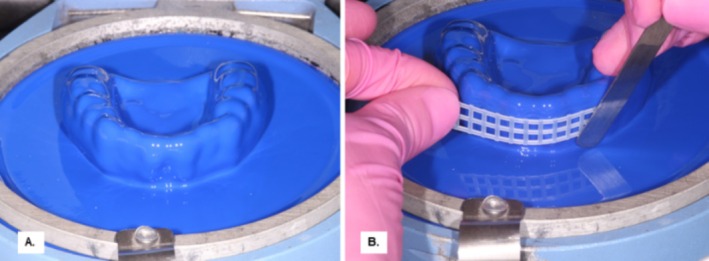
Nylon mesh reinforcement applied to the surface of the initial EVA layer for MG fabrication in the Mg 1 + 3 group using a 3 mm soft EVA sheet. (A) EVA sheet vacuum‐formed on the model; (B) Silica‐reinforced nylon mesh placed on the first EVA layer for reinforced MG fabrication (Mg 1 + 3).

For the reinforced groups (Figures 5 and 6), the nylon mesh reinforcement was placed within the MG along the intercanine surface (Figure 6) before layering sheets of varying thicknesses.

After inserting the mesh, a second layer of soft EVA was added to complete the production of the reinforced MG. Following MG fabrication, the excess material was trimmed from the plaster model, and the cut area was marked with a permanent marker (Pilot, São Paulo, Brazil). The vestibular region was shaped to extend 2 mm below the vestibule fold, while the palatal extension was maintained at 10 mm beyond the gingival margin [30]. The initial cut was made with straight iris scissors, while excess material was removed using Maxi and Minicut burs (Labordental, Indianapolis, USA), followed by finishing procedures with Scotch‐Brite brushes (PM, Reference MSH78WH‐1/American Burs, coarse to extra‐fine grit) and a Hanau torch.

#### 2.2.3 Strain Test

Strain gauges were employed to conduct a mechanical laboratory test aimed at measuring microstrain and impact absorption. The fabricated maxillofacial model was subjected to an impact test using the custom‐made MGs described above, with the non‐reinforced MG serving as the control group.

**FIGURE 7:**
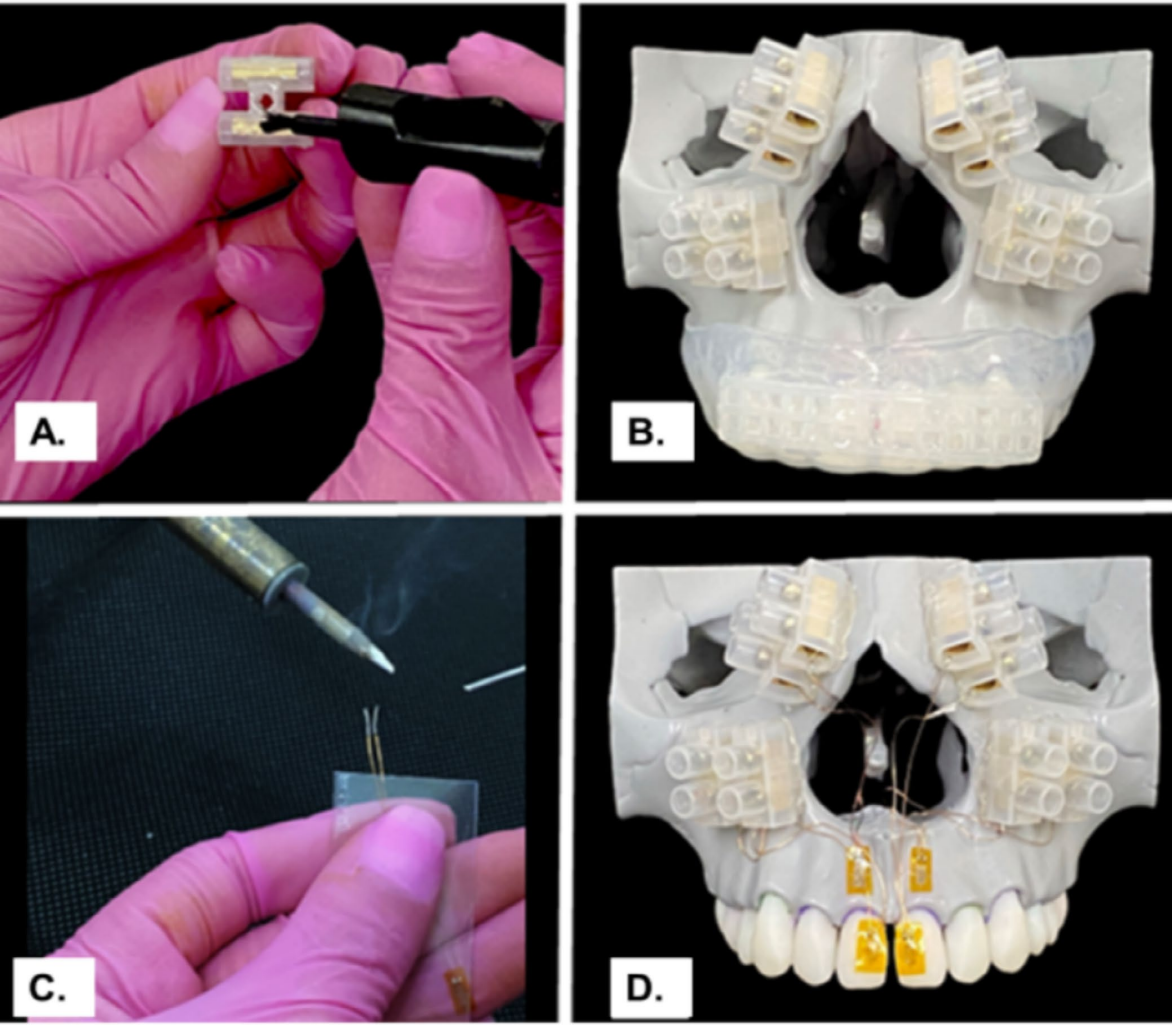
Sequence of the bonding process for connectors and strain gauges for strain test. (A) Electrical connectors bonded with cyanoacrylate; (B) Skull with connectors attached to the pre‐planned location; (C) soldering, the strain gauge ends where no enamel is present; (D) Strain gauges attached with cyanoacrylate in the pre‐planned locations.

In order to accurately capture deformation data, specific preparatory steps were necessary for the maxillofacial model. After cleaning the entire surface of the maxilla model with isopropyl alcohol, a unidirectional strain gauge (PA‐06‐125BA‐120‐L; EXCEL sensors Ind. Com. Exp. LTDA., Brazil, internal resistance 120 Ω; width: 1.5 mm; length: 3 mm; sensitivity/gauge factor 2.14) was adhered using cyanoacrylate glue (Super Bonder Loctite, Henkel Ltda., São Paulo, SP, Brazil) to the central surfaces of the crowns of the upper central incisors (teeth 11 and 21) and the surface of the alveolar process of the maxilla at the root level of the incisors (Figure 7). The strain gauge cables were connected to a data acquisition system, establishing a connection between the measured stress and the recording device. Measurement resistance was verified with a multimeter (Minida ET 2055: Minida São Paulo, Brazil), and changes in electrical resistance were converted into microstrain units through a data transformation and analysis device (Lynx, ADS‐2000, São Paulo, SP, Brazil), with data recorded in Hz.

#### 2.2.4 Impact Testing

**FIGURE 8:**
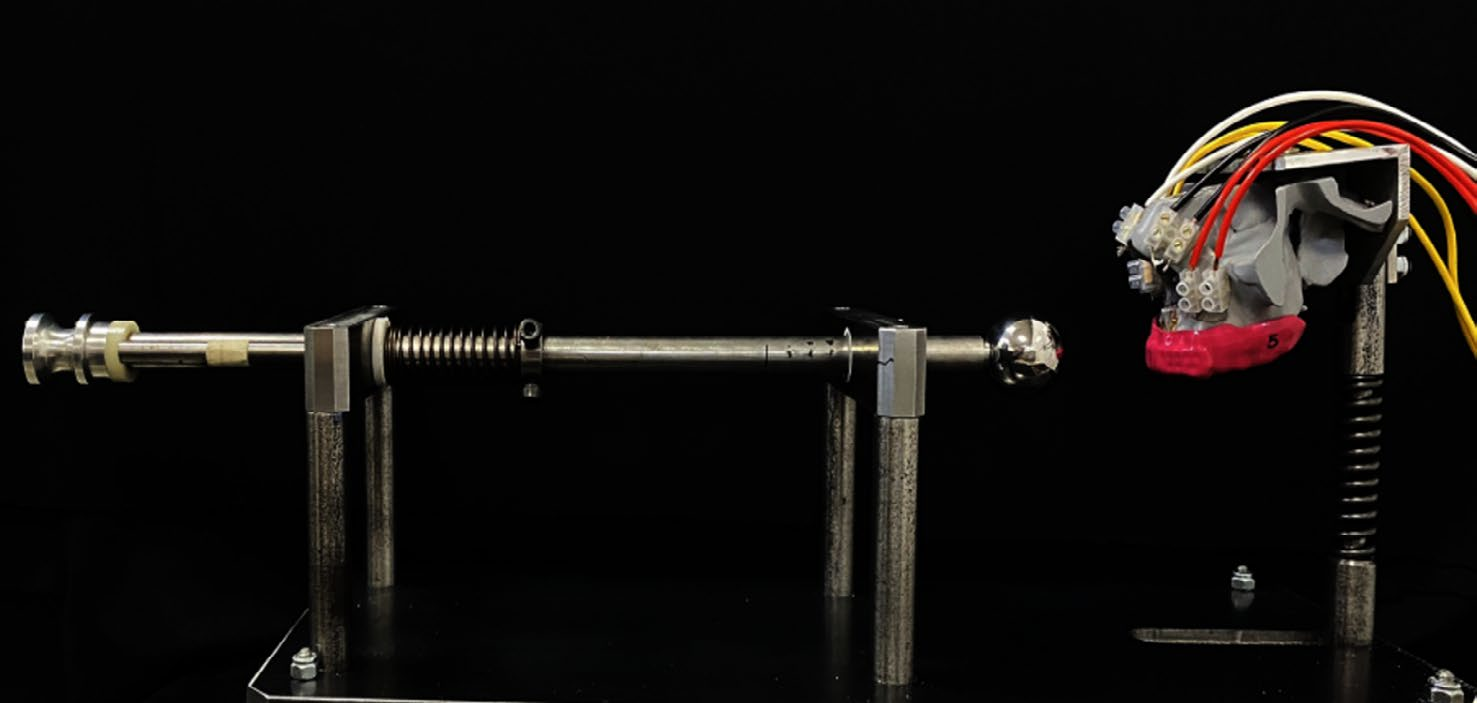
Impact machine created for controlled load application, using a piston with a spring of known elastic constant, deformed over a standardized distance, adjusted by a load application rod.

A specific impact machine was developed (Figure 8) to be used in association with strain analyses. This machine enables a controlled application of impact at a fixed speed and intensity, directed horizontally and parallel to the ground, on the upper central incisor region according to predefined groups to examine dentoalveolar trauma patterns.

The impact was simulated through contact between the properly positioned mouthguard and a 35 mm diameter metal sphere attached to the impact machine. The sphere, attached to a tensioned spring, was always released in a controlled and standardized way, generating a force that produced measurable deformation in the strain gauges fixed to the skull. This force was kept within the elastic limit of the material and below critical stress levels. Tests were conducted in triplicate for each sample of each group.

The spring deformation was defined based on the elastic constant of the spring used, with a value of 687 N/m. The calculation of Elastic Potential Energy (Ep) was applied according to the formula: Ep = *k*·*x* ^2^/2, where “Ep” represents Elastic Potential Energy in Joules, “*k*” the elastic constant (N/m), and “*x*” the spring deformation (m). The use of Elastic Potential Energy calculations was deemed to be appropriate for this study, as it represents the energy stored in an elastic material through the application of force.

The Elastic Potential Energy applied was calculated with the spring stretched to 4 cm, as shown in Equation (1):

(1) $$\mathrm{Ep}=\left(k\cdot x^{2}\right)/2$$

$$\mathrm{Ep}=\left(687\cdot 0.042\right)/2$$

Ep=0.5496J

In order to calculate the elastic force applied, Hooke's Law was applied: Fel = −*k*·*x*, based on the principle that when a force is applied to a spring, it deforms, generating an elastic force with the same direction as the external force but in the opposite direction. The negative sign indicates that the elastic force is opposite to the direction of spring extension, as shown in Equation (2):

(2) Fel=−k·x

Fel=−687·0.04

Fel=−27.48N/m

∣Fel∣=27.48N/m

Given the Elastic Force (Fel) and the mass of the impact application assembly (0.901 kg), acceleration can be calculated using Equation (3):

(3) $$a=\left(k\cdot x\right)/m$$

$$a=\left(687\cdot 0.04\right)/0.901$$

$$a=30.49\;m/s^{2}$$

Assuming an initial velocity of 0 and neglecting air resistance, the uniformly accelerated motion equation (Torricelli's equation), as shown in Equation (4):

(4) $$V^{2}=V_{0}+2\mathrm{a\Delta S}$$

$$V^{2}=0+2\left(30.49\right)\;\left(0.04\right)$$

V=1.56m/s

To determine the time of impact, again neglecting air resistance, Equation (5) was used:

(5) $$V=V_{0}+\mathrm{at}$$

1.56=0+30.49·t

t=0.05s

## 3 Results

The coherence analysis of the mathematical model was conducted by assessing total displacement to verify if the assembly responded consistently with the pre‐established load and fixation parameters. Additionally, it was confirmed that the contacts defined in the preprocessing phase indeed were connected, ensuring that the energy from one body flowed and was properly transferred to the other (von Mises Equivalent stress).

**FIGURE 9:**
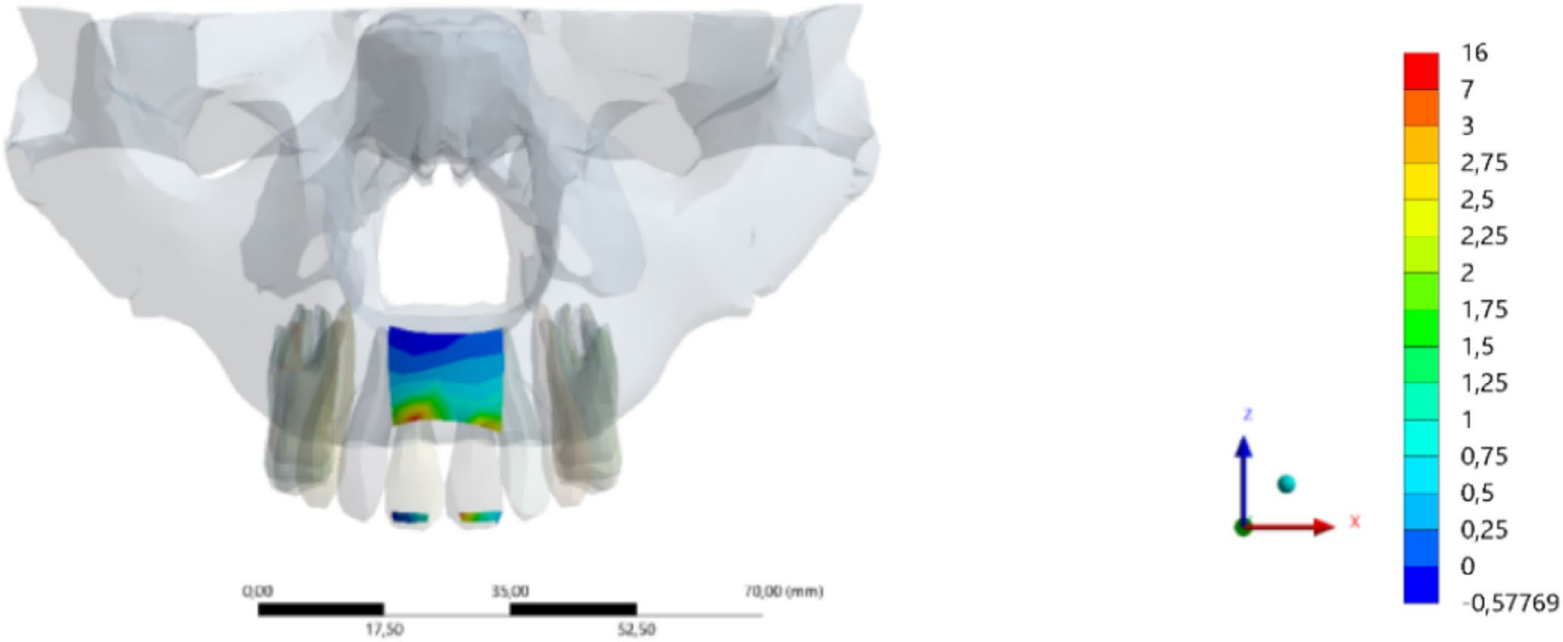
Post‐impact measurement locations in the central incisors and maxillary alveolar process via finite element analysis.

The analysis was conducted at the precise moment when the ball contacted the mouthguard. Equivalent deformations were measured, resulting in the average equivalent elastic deformation in stress concentration regions measured by the strain gauges (microstrain—με), as shown in Figure 9 and Tables 3 and 4.

**TABLE 3:** Microstrain values obtained in the maxillary alveolar process after using different types of mouthguards with and without reinforcement in finite element analysis (in silico study) and strain gauge (in vitro study).

Mouthguard type	FEA touch time	FEA value
Control	0.0089 s	319 με
Mg 1 + 3	0.0063 s	267 με
Mg 2 + 2	0.0063 s	270 με
Mg 3 + 1	0.0063 s	221 με

**TABLE 4:** Microstrain values obtained in the upper central incisors after using different types of mouthguards with and without reinforcement in finite element analysis (in silico study) and strain gauge (in vitro study).

Mouthguard type	FEA touch time	FEA value
Control	0.0089 s	524 με
Mg 1 + 3	0.0063 s	124 με
Mg 2 + 2	0.0063 s	114 με
Mg 3 + 1	0.0063 s	119 με

The data showed that the control group presented a higher concentration of stress compared to the other groups. However, no relevant differences were observed among the reinforced groups.

### 3.1 Statistical Analysis

The experimental variables studied were: Group—“Mouthguard—MG” (polychotomous nominal qualitative variable—Control, Mg 1 + 3, Mg 2 + 2, Mg 3 + 1), Impact Absorption Area—“Area” (dichotomous nominal qualitative variable—Alveolar bone and tooth), and Deformation—“Strain” (continuous quantitative variable). Statistical tests, including the normality test (Shapiro–Wilk) and non‐parametric tests (Kruskal–Wallis and Dunn's test), were conducted using R‐project software version 3.2.0. The significance level was set at 5%, establishing a 95% confidence interval for the presented results.

After data analysis, normality tests (Shapiro–Wilk) were performed for deformation values, yielding a value of 0.825 and a *p*‐value of 1.5 × 10^−12^. With *p*‐values < 0.05 for the Shapiro–Wilk test, it was determined that the strain distribution is non‐normal, indicating the use of non‐parametric tests.

Results for the peripheral microstrain analysis of the different mouthguard types were obtained, with respective values absorbed by the dental elements and alveolar bone. Table 5 presents the microstrain modulus values obtained from the strain test for the evaluated groups.

**TABLE 5:** Mean ± standard deviation (SD) of microstrain (με) for different mouthguards.

Group	Tooth	Alveolar bone	Total
Control	122.4 (92.6)	165.7 (106.1)	144.0 (100.7)
Mg 1 + 3	88.3 (60.1)	73.1 (60.1)	78.5 (53.1)
Mg 2 + 2	77.5 (73.0)	90.6 (72.5)	85.2 (72.0)
Mg 3 + 1	77.1 (64.7)	58.3 (33.0)	67.7 (51.6)

For a detailed analysis of the results, a Kruskal–Wallis statistical test was applied to evaluate the relationship of the “Group” variable with its deformation values. Additionally, Dunn's multiple comparison test was applied to interpret the results more precisely. The results obtained showed a statistically significant difference (*p* = 6.8 × 10^−5^), with higher deformations for the “Control” group (Table 6).

**TABLE 6:** Kruskal–Wallis and Dunn test results for the “Group” factor.

Test data	με (SD)	DF	*X* ^2^	*p*
Control	144.0 ± 100.7 ^A^	3	21.8	< 0.00000
Mg 1 + 3	78.5 ± 53.1 ^B^			
Mg 2 + 2	85.2 ± 72.0 ^B^			
Mg 3 + 1	67.7 ± 51.6 ^B^			

*Note:* Uppercase letters indicate comparison between columns; different letters indicate *p* < 0.05.

Analyzing the microstrain (με) in the different impact areas, no statistical difference was observed (*p* = 0.9679) (Table 7).

**TABLE 7:** Mann–Whitney table for the “Area” Variable.

Test data	με (SD)	W	*p*
Alveolar bone	90.6 ± 72.2	3212	0.967
Tooth	96.9 ± 82.6		

**FIGURE 10:**
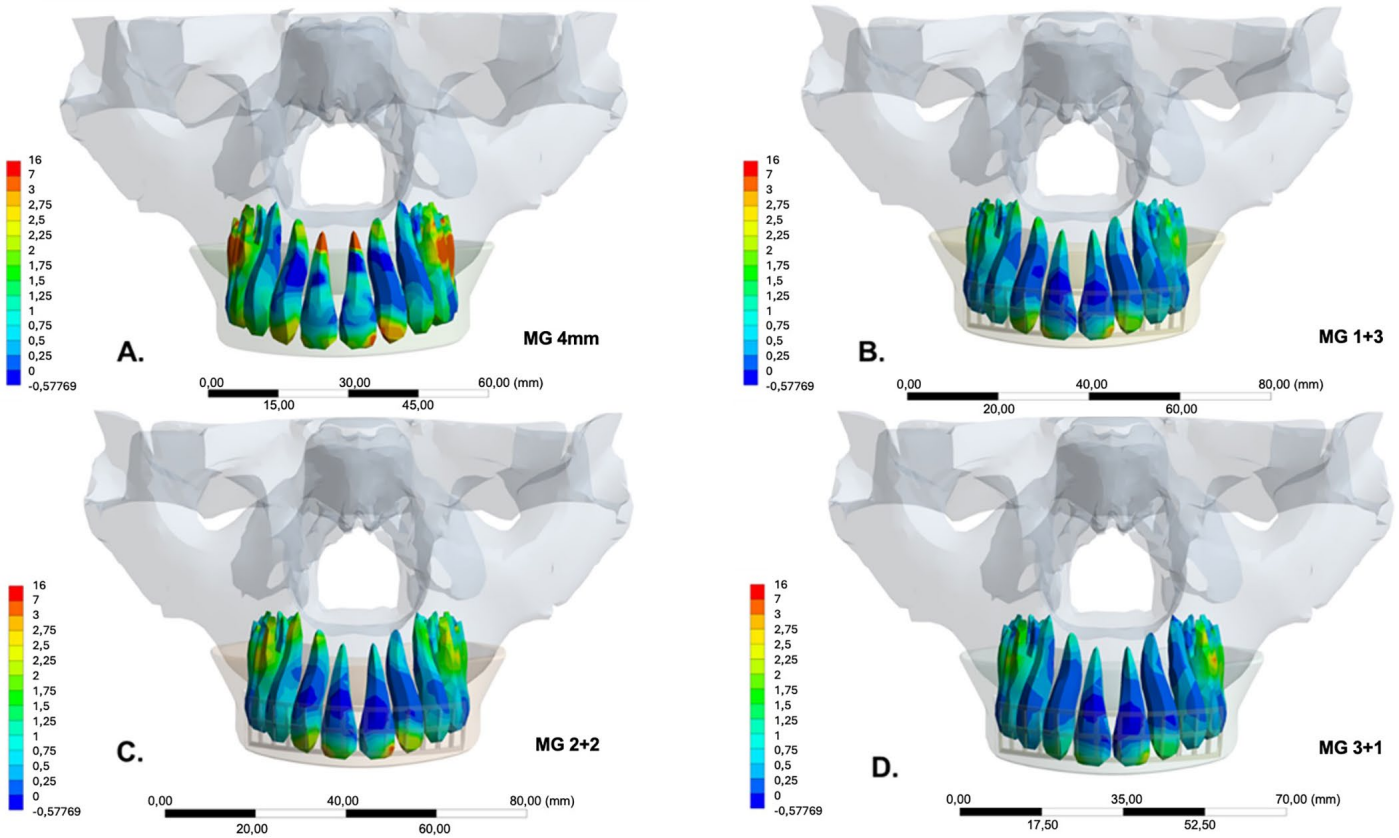
Maximum Principal Stress (MPa) in upper central incisors post‐impact with different types of mouthguards. (A) Conventional, non‐reinforced mouthguard (control). (B) Mouthguard with reinforcement 1 mm from the vestibular limit (MG 1 + 3). (C) Mouthguard with reinforcement 2 mm from the vestibular limit (MG 2 + 2). (D) Mouthguard with reinforcement 3 mm from the vestibular limit (MG3 + 1).

The results obtained from the different methodologies (in vitro and in silico studies) show proportional results to each other, which infers that the mathematical model and the in vitro analysis are compatible, indicating methodological validation. Thus, the mathematical model for measurement studies with complex structures was capable of identifying impacted regions and the biomechanical behavior of the entire maxillofacial complex. Furthermore, the concentration of stress from impact absorption, as previously quantified, was visually represented via finite element analysis in Maximum Principal Stress (MPa) (Figure 10). The results show a clear distinction between the conventional non‐reinforced group and the reinforced groups (MG 1 + 3, MG 2 + 2, and MG 3 + 1), with no significant differences between the reinforced groups.

The colorimetric scale presented below indicates the areas of greatest stress concentration. Warmer colors predominate in the model with the non‐reinforced MG, with the highest concentration at the root apex of the upper central incisors, as well as at the disto‐incisal region of tooth 21 and the mesio‐incisal area of tooth 22. This difference may be due to the lack of complete anatomical symmetry, potentially resulting in millimeter‐sized protrusions. The reinforced groups exhibited no significant differences; however, a minor increase in stress concentration was observed in the disto‐incisal region of tooth 21 in the MG 2 + 2 group. This may be explained by the mesh being situated in the neutral zone relative to compressed and tensile areas, but these characteristics are similar to the MG 1 + 3 group. It is notable that the colorimetric characteristics and numerical data presented by the finite element analysis are consistent with the strain gauge findings.

## 4 Discussion

Advances in sports dentistry emphasize the critical need for innovative approaches in the development of mouthguards that enhance energy absorption and dissipation [31]. Although custom‐made mouthguards meeting established standards for thickness and structural parameters are widely used, limitations in their protective efficacy remain, as evidenced by continued reports of orofacial injuries, especially involving the central incisors [32, 33]. It enhances the importance of investigating reinforcement strategies for ethylene vinyl acetate (EVA) mouthguards to achieve more comprehensive and effective energy distribution during impacts [34].

A systematic review by Shelley and colleagues [34] examined the protective capacity of anterior teeth when reinforced mouthguards were used. The review included twelve studies, in which eleven were laboratory‐based: eight employing pendulum impact testing [13, 32, 35, 36, 37, 38, 39] and three using free‐weight drop devices [8, 40, 41]. Additionally, a theoretical study using finite element analysis (FEA) [42] was included. The review found that methodological quality in these studies ranged from low to intermediate, with impact energies considerably lower than real‐world conditions, and that the test specimens were made from materials that lacks biological fidelity. Although studies by Bochnig et al. (2017), Handa et al. (2011), Matsuda et al. (2020), Oikarinen et al. (1993), Sarac et al. (2019), Takeda et al. (2006), and Watermeyer et al. (1985) demonstrated benefits with rigid reinforcements in mouthguards, conclusive evidence regarding their efficacy remains limited [10, 13, 32, 33, 37, 39, 41].

This study shows that the effectiveness of reinforcement material depends on its inclusion and placement within the mouthguard, though placement location is not critical. The study utilized a methodology that closely replicates controlled loading conditions and featured test specimens designed to closely approximate anatomical reality within the limitations inherent to in vitro studies.

The primary metric outcome in impact testing is the energy absorption of the specimen during deformation or fracture. However, the Charpy pendulum impact test, a common method, has limitations, primarily because it does not reliably capture stress in notched regions due to variations among different materials. This limits the test's use to qualitative analysis and comparisons within identical materials [43]. Additionally, the Charpy test is limited in a way that it primarily measures energy absorbed at fracture, which may differ significantly from real‐world conditions [44]. Weight‐drop testing has been shown to induce fractures at loads within the material's elastic behavior [43], though it does not provide precise speed control.

Given the need for a more realistic assessment of dynamic loading conditions, a specialized impact apparatus was developed in this study, enabling controlled impact application with standardized speed and intensity. This design was specifically chosen to address the high incidence of trauma in the anterior dental region [8, 32]. This impact apparatus standardized parameters for elastic potential energy at 0.5 J, applied elastic force at 27.5 N/m, acceleration at 30.5 m/s^2^, velocity at 1.5 m/s, and impact duration at 0.05 s across all samples. Used in conjunction with a strain gauge, this apparatus quantified differences in impact response among various mouthguard designs, allowing for high‐sensitivity quantification during material deformation. Strain gauge sensors provided accurate measurements for both static and dynamic loads, with calculations based on resistance variation translated into strain levels by considering the ratio between length change and original length [45]. This approach allowed maximum deformations to be captured at the moment of impact, with FEA providing complementary data under similar conditions for proper validation.

Given the diversity of materials and anatomical designs in laboratory studies, as well as the limited biological fidelity of these models [34], a complex skull model was created with strategic flat cuts in the nasal, occipital, and zygomatic bone regions for secure fixation within the impact apparatus. Neck simulation was achieved using a rigid spring, which represented natural post‐impact movement, similar to the model proposed by de Wet et al. (1999) [36]. Unlike studies that employed rigid maxillary stabilization (e.g., Greasley et al., 1998) [40], this model allowed for posterior‐inferior support movement under impact, closely resembling the mobile joint behavior from the neck.

In order to prepare the skull for analysis, specimens were 3D‐printed using resins with mechanical properties comparable to those previously described for polyurethane resin (3.6 GPa) and medullary bone (4.0–4.5 GPa), ensuring stimulus response fidelity [46, 47]. In this study, Spin Red resin from Quanton 3D, with an elastic modulus of 3.42 GPa and a Poisson ratio of 0.56, was used as a substitute for 3D‐unprintable polyurethane resin. This choice enabled specimen fabrication with sufficient impact energy to activate strain gauges without surpassing the material's elastic limit, thereby avoiding fractures or permanent deformations that could interfere with the results.

The skull model comprised a printed skull with teeth anchored by alveolus, a simulated periodontal ligament made of elastomer, and was mounted on a structure that partially simulated neck movement, closely approximating in vivo conditions. Despite the model's innovation and the boundary conditions established in this study, limitations remain, including the absence of soft tissue cushioning, a limitation also encountered in de Wet et al. (1999) study [36].

An important aspect to consider is the low impact energy achieved, measured at 0.549 J. While this value is considerably lower than the kinetic energy typically generated by a baseball (approximately 205 J) or an ice hockey puck (185 J) [34], it aligns with previous studies that have reported maximum impact energy of 0.00137 J in dynamic non‐linear analysis at 1 m/s via FEA [42]; 0.388 J in free‐weight drop impact tests [41]; and studies employing pendulum impact testing with energies of 0.16 J [36], 0.23 J [35], 0.148 J [10], 0.148 J [33], and 0.158 J [13]. In contrast, higher energy values were reported in studies by Watermeyer et al. (1985) [39], Bochnig et al. (2017) [32], and Sarac et al. (2019) [37], with maximum energy levels of 0.945 J and 1.719 J, respectively, in pendulum tests. Free‐weight drop tests by Greasley et al. (1998) [40] and Kataoka et al. (2014) [8] reported energy levels of 10.002 J and 5.585 J, respectively. Thus, while the experimental impact energy in this study was lower than in some others, future studies are needed to determine a more realistic energy threshold for orofacial trauma analysis.

The ideal custom mouthguard thickness is well‐established at 3–4 mm [8, 31, 48]. However, due to the high incidence of orofacial injuries, various studies have examined reinforced mouthguards [10, 33, 49]. Rigid reinforcements are suggested because they disperse stress across adjacent structures, preventing localized concentrations [34]. Reinforced mouthguards have incorporated materials like air cells, sorbothane, orthodontic metal wires, sponges, lead sheets, and acrylic plates [8, 10, 13], as well as alternative polymers such as polyamides, polyethylene terephthalate glycol (PETG) and thermoplastic polyurethane (TPU) [38]. However, the optimal reinforcement material remains undetermined, and limitations persist regarding costs, biofidelity, and clinical applicability [33].

The results of this study rejected the first null hypothesis, as the reinforced mouthguards showed significantly improved impact absorption compared to the control (*p* < 0.05). However, the lack of significant differences between the reinforced groups (Mg 1 + 3; Mg 2 + 2; Mg 3 + 1) (*p* > 0.05) supports the second hypothesis, indicating that the placement of reinforcement within the mouthguard did not affect impact absorption. No significant differences were observed in impact absorption between alveolar bone and central incisor regions (*p* > 0.05), demonstrating that EVA‐reinforced mouthguards provide effective protection across different anatomical sites.

The finite element analysis findings were consistent with in vitro results, which conventional mouthguards had the lowest stress absorption capacity following impact, whereas reinforced mouthguards—regardless of mesh placement—demonstrated a more favorable stress distribution. Moreover, the in vitro model validated the mathematical model, supporting its use in future complex analyses, including evaluations of facial bones, periodontal ligaments, and other structurally intricate regions that are challenging to analyze in vivo due to the constraints of impact testing and the methodological limitations associated with in vitro studies.

This study aligns with existing literature when suggesting that reinforced mouthguards may offer viable alternatives to conventional designs. A study by Handa et al. (2011) demonstrated that EVA mouthguards with a 3 mm thickness, increased by 1 mm of acrylic resin and 1.8 mm of konbiplastic (EVA + PETG), achieved up to 90% impact absorption, while unreinforced EVA mouthguards absorbed only 55%–78% [33]. Takeda et al. (2006) observed similar results, finding that mouthguards reinforced with fiberglass and resin provided superior protection compared to bilaminated EVA designs [10]. Additional impact absorption reinforcement methods, such as the inclusion of air cavities, have been shown to reduce impact by 32% [10], while an intermediate sorbothane layer decreased impact transmission by approximately 30% [33]. The use of sponge between two EVA layers reduced impact by 49%, [10] and an A‐silicone intermediary layer improved impact absorption by 15%–25% compared to conventional mouthguards [50].

In contrast, a previous study by Kataoka et al. (2014) used a model including bone, tooth, and gingiva with a 4 mm thick mouthguard reinforced with 2 mm of Titanium that showed no significant differences in impact reduction between reinforced and unreinforced mouthguards during frontal impact from a free‐weight drop test with a 1.7 kg mass [8]. This lack of difference may be attributed to elastic limit exceedance in the materials tested, where energy levels were sufficient to cause damage independent of mouthguard reinforcement.

This study aimed to fill the knowledge gap by integrating in vitro and in silico methods under experimentally realistic conditions, as suggested by other studies to be necessity [34]. The explicit nonlinear dynamic analysis was used to simulate impact, incorporating speed data from the in vitro study. This approach was essential because the most valid method of assessing the accuracy of these methodologies is through complementary data associations that confirm result compatibility [45]. This way, we can simulate a different arc form, tooth position, and anatomies to visualize the stress distribution in different situations of force application, since this kind of study is not reproductive in vivo. These findings and the validation of the in silico model open up possibilities for further in‐depth studies. The results of the present study can provide incentives for the clinical field to implement significant improvements in the quality of sports mouthguards, as it employs a strategy capable of enhancing impact absorption in these devices through the inclusion of reinforcements. Thus, the incorporation of a nylon mesh between the layers of the device serves as a means to further reduce the issue of these injuries [51], which negatively affect athletes by keeping them away from training and competitions, consequently impacting their relationships with sponsors as well as their emotional well‐being [52, 53].

Limitations of the present study can be observed, including the absence of oral cavity conditions, such as pH variation, moisture, and temperature fluctuations [54, 55]; the low impact energy employed; and the lack of soft tissue cushioning. Additionally, an ideal occlusion was assumed, with no adaptation issues and impact restricted to the mouthguard region [56].

## 5 Conclusion

Based on the findings of this study:

- The addition of reinforcement systems to mouthguards enhanced their protective efficacy
- However, the location of the reinforcement within the mouthguard does not significantly influence impact absorption in oral tissues;
- The finite element analysis results corroborate the in vitro strain‐gauge findings, thereby validating the numerical model and demonstrating that both methodologies are applicable for biomechanical studies of trauma.

## Author Contributions

Conceptualization: T.S.d.Q. and A.L.S.B.; Methodology: T.S.d.Q, L.H.e.B., T.J.d.A.P.J., A.L.S.B. and J.P.M.T.; Validation: A.L.S.B. and J.P.M.T.; Formal analysis: A.L.S.B., G.d.R.S.L., and J.P.M.T.; Investigation: T.S.d.Q., L.H.e.B., and A.L.S.B.; Data curation, T.S.d.Q., A.L.S.B., and G.R.S.L.; Writing – original draft preparation: T.S.d.Q.; Writing – review and editing: T.S.d.Q., L.H.e.B., A.L.S.B., J.P.M.T., and T.J.d.A.P.J.; Visualization: T.S.d.Q., A.L.S.B. and L.H.B.; Supervision: A.L.S.B., J.P.M.T. and T.J.d.A.P.J.; Project administration: A.L.S.B., J.P.M.T., and T.J.d.A.P.J.; Funding acquisition: A.L.S.B., J.P.M.T. and T.J.d.A.P.J. All authors have read and agreed to the published version of the manuscript.

## Conflicts of Interest

The authors declare no conflicts of interest.

## Data Availability

Research data are not shared.

## References

[1] J. P. Tribst , A. M. Dal Piva , P. C. de Carvalho , P. H. Gonçalves , A. L. Borges , and T. J. Paes‐Junior , “Does Silica–Nylon Mesh Improve the Biomechanical Response of Custom‐Made Mouthguards?,” Sport Sciences for Health 16 (2020): 75–84.

[2] J. P. Tribst , A. M. Dal Piva , M. A. Bottino , C. J. Kleverlaan , and J. H. Koolstra , “Mouthguard Use and TMJ Injury Prevention With Different Occlusions: A Three‐Dimensional Finite Element Analysis,” Dental Traumatology 36, no. 6 (2020): 662–669. 32460432 10.1111/edt.12577

[3] H. K. Park , J. Y. Park , N. R. Choi , U. K. Kim , and D. S. Hwang , “Sports‐Related Oral and Maxillofacial Injuries: A 5‐Year Retrospective Study, Pusan National University Dental Hospital,” Journal of Oral and Maxillofacial Surgery 79, no. 1 (2021): 203.e1–203.e8. 10.1016/j.joms.2020.07.218 32866487

[4] M. Saito , K. Nakajima , A. Tsutsui , et al., “Effects of Mouthguards on Skin Damage: An In Vitro Study,” European Journal of Dentistry 17, no. 3 (2022): 740–748. 36307114 10.1055/s-0042-1756474 PMC10569882

[5] S. K. Sarao and L. Levin , “Prevention of Maxillofacial Injuries Through Analysis of Mechanisms, Patterns, and Long‐Term Sequelae,” Dental Traumatology 39, no. 2 (2023): 97–100. 36899141 10.1111/edt.12833

[6] E. B. Tuna and E. Ozel , “Factors Affecting Sports‐Related Orofacial Injuries and the Importance of Mouthguards,” Sports Medicine 44 (2014): 777–783. 24647854 10.1007/s40279-014-0167-9

[7] L. M. Fernandes , J. C. Neto , T. F. Lima , et al., “The Use of Mouthguards and Prevalence of Dento‐Alveolar Trauma Among Athletes: A Systematic Review and Meta‐Analysis,” Dental Traumatology 35, no. 1 (2019): 54–72. 30222244 10.1111/edt.12441

[8] S. H. Kataoka , F. C. Setzer , E. Gondim, Jr. , and C. L. Caldeira , “Impact Absorption and Force Dissipation of Protective Mouthguards With or Without Titanium Reinforcement,” Journal of the American Dental Association (1939) 145, no. 9 (2014): 956–959. 25170003 10.14219/jada.2014.54

[9] J. P. Tribst , A. M. de Oliveira Dal Piva , A. L. Borges , and M. A. Bottino , “Influence of Custom‐Made and Stock Mouthguard Thickness on Biomechanical Response to a Simulated Impact,” Dental Traumatology 34, no. 6 (2018): 429–437. 30107079 10.1111/edt.12432

[10] T. Takeda , K. Ishigami , J. Handa , et al., “Does Hard Insertion and Space Improve Shock Absorption Ability of Mouthguards?,” Dental Traumatology 22, no. 2 (2006): 77–82. 16499630 10.1111/j.1600-9657.2006.00361.x

[11] Y. R. Bulsara and I. R. Matthew , “Forces Transmitted Through a Laminated Mouthguard Material With a Sorbothane Insert,” Dental Traumatology 14, no. 1 (1998): 45–47. 10.1111/j.1600-9657.1998.tb00807.x 9643177

[12] D. Patrick , R. F. Van Noort , and M. S. Found , “Evaluation of Laminated Structures for Sports Mouthguards,” Key Engineering Materials 221 (2001): 133–144.

[13] Y. Matsuda , K. Nakajima , M. Saitou , et al., “The Effect of Light‐Cured Resin With a Glass Fiber Net as an Intermediate Material for Hard & Space Mouthguards,” Dental Traumatology 36, no. 6 (2020): 654–661. 32304262 10.1111/edt.12560

[14] T. J. Paes‐Junior , H. L. De Castro , A. L. Borges , A. Della Bona , and F. D. Gonçalves , “A Novel Silica‐Nylon Mesh Reinforcement for Dental Prostheses,” Advances in Materials Science and Engineering 2017 (2017): 1–6.

[15] P. Cevik and A. Z. Yildirim‐Bicer , “The Effect of Silica and Prepolymer Nanoparticles on the Mechanical Properties of Denture Base Acrylic Resin,” Journal of Prosthodontics 27, no. 8 (2018): 763–770. 27898997 10.1111/jopr.12573

[16] C. S. Almeida , M. Amaral , F. D. Gonçalves , and T. J. de Arruda Paes‐Junior , “Effect of an Experimental Silica‐Nylon Reinforcement on the Fracture Load and Flexural Strength of Bisacrylic Interim Partial Fixed Dental Prostheses,” Journal of Prosthetic Dentistry 115, no. 3 (2016): 301–305. 26548883 10.1016/j.prosdent.2015.08.009

[17] T. J. Paes‐ Junior and M. Amaral , “Stress Distribution of Complete‐Arch Implant‐Supported Prostheses Reinforced With Silica‐Nylon Mesh,” Journal of Clinical and Experimental Dentistry 11, no. 12 (2019): e1163. 31824598 10.4317/jced.56470 PMC6894919

[18] A. L. Borges , A. M. Dal Piva , L. R. Concílio , T. J. Paes‐Junior , and J. P. Tribst , “Mouthguard Use Effect on the Biomechanical Response of an Ankylosed Maxillary Central Incisor During a Traumatic Impact: A Three‐Dimensional Finite Element Analysis,” Life (Basel) 10, no. 11 (2020): 294. 33233499 10.3390/life10110294 PMC7699499

[19] J. J. Knapik , S. W. Marshall , R. B. Lee , et al., “Mouthguards in Sport Activities: History, Physical Properties, and Injury Prevention Effectiveness,” Sports Medicine 37 (2007): 117–144. 17241103 10.2165/00007256-200737020-00003

[20] Z. Sun , J. Zhang , R. Sun , et al., “Effects of Different Custom‐Made Mouthguard Palatal Extensions on the Stress‐State of Dentoalveolar Structures: A 3D‐FEA,” Clinical Oral Investigations 27, no. 7 (2023): 3809–3816. 37010637 10.1007/s00784-023-04998-0

[21] H. Sano , T. Shono , H. Sonoda , et al., “Relationship Between Surface Area for Adhesion and Tensile Bond Strength—Evaluation of a Micro‐Tensile Bond Test,” Dental Materials 10, no. 4 (1994): 236–240. 7664990 10.1016/0109-5641(94)90067-1

[22] J. S. Rees and P. H. Jacobsen , “Elastic Modulus of the Periodontal Ligament,” Biomaterials 18, no. 14 (1997): 995–999. 9212195 10.1016/s0142-9612(97)00021-5

[23] A. Tsouknidas , E. Karaoglani , N. Michailidis , D. Kugiumtzis , A. Pissiotis , and K. Michalakis , “Influence of Preparation Depth and Design on Stress Distribution in Maxillary Central Incisors Restored With Ceramic Veneers: A 3D Finite Element Analysis,” Journal of Prosthodontics 29, no. 2 (2020): 151–160. 31663223 10.1111/jopr.13121

[24] C. Holberg , A. K. Heine , P. Geis , K. Schwenzer , and I. Rudzki‐Janson , “Three‐Dimensional Soft Tissue Prediction Using Finite Elements,” Journal of Orofacial Orthopedics 66, no. 2 (2005): 122–134. 15827700 10.1007/s00056-005-0422-7

[25] C. Verissimo , P. V. Costa , P. C. Santos‐Filho , et al., “Custom‐Fitted EVA Mouthguards: What Is the Ideal Thickness? A Dynamic Finite Element Impact Study,” Dental Traumatology 32, no. 2 (2016): 95–102. 26310199 10.1111/edt.12210

[26] A. S. Firmino and R. N. Tango , “Analysis of Bond Strength Between a Nylon Reinforcement Structure and Dental Resins,” Journal of Clinical and Experimental Dentistry 13, no. 5 (2021): e505. 33981399 10.4317/jced.57654 PMC8106934

[27] M. Miyashiro , V. Suedam , R. T. Moretti Neto , P. M. Ferreira , and J. H. Rubo , “Validation of an Experimental Polyurethane Model for Biomechanical Studies on Implant‐Supported Prosthesis‐Tension Tests,” Journal of Applied Oral Science 19 (2011): 244–248. 21625741 10.1590/S1678-77572011000300012 PMC4234337

[28] A. C. Souza , T. A. Xavier , J. A. Platt , and A. L. S. Borges , “Effect of Base and Inlay Restorative Material on the Stress Distribution and Fracture Resistance of Weakened Premolars,” Operative Dentistry 40, no. 4 (2015): E158–E166. 25764042 10.2341/14-229-L

[29] A. L. Borges , A. L. S. Borges , A. M. O. Dal Piva , L. R. D. S. Concílio , T. J. A. Paes‐Junior , and J. P. M. Tribst , “Mouthguard Use Effect on the Biomechanical Response of an Ankylosed Maxillary Central Incisor During a Traumatic Impact: A 3‐Dimensional Finite Element Analysis,” Life (Basel) 10, no. 11 (2020): 294, 10.3390/life10110294. 33233499 PMC7699499

[30] A. M. Sousa , A. C. Pinho , A. Messias , and A. P. Piedade , “Present Status in Polymeric Mouthguards: A Future Area for Additive Manufacturing?,” Polymers (Basel) 12, no. 7 (2020): 1490. 32635307 10.3390/polym12071490 PMC7407806

[31] D. A. Mendel , Y. Ucar , W. A. Brantley , R. G. Rashid , S. L. Harrell , and T. H. Grentzer , “Impact Energy Absorption of Three Mouthguard Materials in an Aqueous Environment,” Dental Traumatology 25, no. 1 (2009): 130–135. 19208026 10.1111/j.1600-9657.2008.00751.x

[32] M. S. Bochnig , M. J. Oh , T. Nagel , F. Ziegler , and P. G. Jost‐Brinkmann , “Comparison of the Shock Absorption Capacities of Different Mouthguards,” Dental Traumatology 33, no. 3 (2017): 205–213. 28231638 10.1111/edt.12324

[33] J. Handa , T. Takeda , K. Kurokawa , T. Ozawa , K. Nakajima , and K. Ishigami , “Influence of Pre‐Laminated Material on Shock Absorption Ability in Specially Designed Mouthguard With Hard Insert and Space,” Journal of Prosthodontic Research 55, no. 4 (2011): 214–220. 21444261 10.1016/j.jpor.2011.02.003

[34] A. Shelley , K. Winwood , T. Allen , and K. Horner , “Effectiveness of Hard Inserts in Sports Mouthguards: A Systematic Review,” British Dental Journal 1 (2022): 9. 10.1038/s41415-022-4089-x 35379927

[35] P. Bemelmanns and P. Pfeiffer , “Shock Absorption Capacities of Mouthguards in Different Types and Thicknesses,” International Journal of Sports Medicine 22, no. 2 (2001): 149–153. 11281619 10.1055/s-2001-11342

[36] F. A. de Wet , M. Heyns , and J. Pretorius , “Shock Absorption Potential of Different Mouth Guard Materials,” Journal of Prosthetic Dentistry 82, no. 3 (1999): 301–306. 10479256 10.1016/s0022-3913(99)70084-3

[37] R. Sarac , J. Helbig , J. Dräger , and P. G. Jost‐Brinkmann , “A Comparative Study of Shock Absorption Capacities of Custom‐Fabricated Mouthguards Using a Triangulation Sensor,” Materials (Basel) 12, no. 21 (2019): 3535. 31661939 10.3390/ma12213535 PMC6862432

[38] T. Takeda , K. Ishigami , T. Ogawa , et al., “Are all Mouthguards the Same and Safe to Use? The Influence of Occlusal Supporting Mouthguards in Decreasing Bone Distortion and Fractures,” Dental Traumatology 20, no. 3 (2004): 150–156. 15144446 10.1111/j.1600-4469.2004.00247.x

[39] G. J. Watermeyer , C. J. Thomas , and C. H. Jooste , “The Protective Potential of Mouthguards,” Journal of the Dental Association of South Africa 40, no. 4 (1985): 173–177. 3864292

[40] A. Greasley , G. Imlach , and B. Karet , “Application of a Standard Test to the In Vitro Performance of Mouthguards,” British Journal of Sports Medicine 32, no. 1 (1998): 17–19. 9562158 10.1136/bjsm.32.1.17 PMC1756058

[41] K. S. Oikarinen , M. A. Salonen , and J. Korhonen , “Comparison of the Guarding Capacities of Mouth Protectors,” Endodontics & Dental Traumatology 9, no. 3 (1993): 115–119. 8243343 10.1111/j.1600-9657.1993.tb00262.x

[42] C. Verissimo , P. C. Santos‐Filho , D. Tantbirojn , A. Versluis , and C. J. Soares , “Modifying the Biomechanical Response of Mouthguards With Hard Inserts: A Finite Element Study,” American Journal of Dentistry 28, no. 2 (2015): 116–120. 26087579

[43] K. E. van Vliet , C. J. Kleverlaan , F. Lobbezoo , J. de Lange , and A. J. van Wijk , “Maximum Impact Heights of Currently Used Mouthguards in Field Hockey,” Dental Traumatology 36, no. 4 (2020): 427–432. 31880846 10.1111/edt.12538 PMC7508175

[44] E. Lucon , “Cost‐Effective Alternatives to Conventional Charpy Tests for Measuring the Impact Toughness of Very‐High‐Toughness Steels,” Journal of Pressure Vessel Technology 140, no. 2 (2018): PVT171145. 10.1115/1.4038902 PMC5992621 29892136

[45] C. Verissimo , P. V. Costa , P. C. Santos‐Filho , et al., “Evaluation of a Dentoalveolar Model for Testing Mouthguards: Stress and Strain Analyses,” Dental Traumatology 32, no. 1 (2016): 4–13. 26139006 10.1111/edt.12197

[46] M. J. Smith , S. James , T. Pover , et al., “Fantastic Plastic? Experimental Evaluation of Polyurethane Bone Substitutes as Proxies for Human Bone in Trauma Simulations,” Legal Medicine (Tokyo, Japan) 17, no. 5 (2015): 427–435. 26130519 10.1016/j.legalmed.2015.06.007

[47] C. E. Datte , “The Influence of Restorative Material, Bone Height and Implant System on the Stress Distribution of Implant‐Supported Posterior Crowns,” International Journal of Development Research 11, no. 2 (2021): 44925–44931.

[48] N. K. Cummins and I. R. Spears , “The Effect of Mouthguard Design on Stresses in the Tooth‐Bone Complex,” Medicine and Science in Sports and Exercise 34, no. 6 (2002): 942–947. 12048319 10.1097/00005768-200206000-00006

[49] J. P. Tribst , A. M. Dal Piva , P. Ausiello , A. De Benedictis , M. A. Bottino , and A. L. Borges , “Biomechanical Analysis of a Custom‐Made Mouthguard Reinforced With Different Elastic Modulus Laminates During a Simulated Maxillofacial Trauma,” Craniomaxillofacial Trauma & Reconstruction 14, no. 3 (2021): 254–260. 34471482 10.1177/1943387520980237 PMC8385621

[50] A. A. Kamenskikh , T. N. Ustjugova , and A. G. Kuchumov , “Modelling of the Tooth Contact Through One‐Layered Mouthguard,” Journal of Physics Conference Series 1129, no. 1 (2018): 12014.

[51] T. S. De Queiroz , B. S. da Cruz , A. M. M. Demachkia , A. L. S. Borges , J. P. M. Tribst , and T. J. A. Paes Junior , “Ergonomic Sports Mouthguards: A Narrative Literature Review and Future Perspectives,” Applied Sciences 13, no. 20 (2023): 11353.

[52] W. M. Van Hout , E. M. Cann , J. H. Abbink , and R. Koole , “An Epidemiological Study of Maxillofacial Fractures Requiring Surgical Treatment at a Tertiary Trauma Centre Between 2005 and 2010,” British Journal of Oral & Maxillofacial Surgery 51, no. 5 (2013): 416–420. 23218202 10.1016/j.bjoms.2012.11.002

[53] C. Zacca , “Investigação da Prevalência de Traumatismos Dento‐Faciais em Praticantes de Boxe e a Importância dos Protetores Bucais nas Consequências dos Traumas [Dissertação],” (2006), Belém: Universidade Federal do Pará, Curso de Odontologia.

[54] A. Darvizeh , S. Tecco , A. Golbaf , A. Beraldi , E. F. Gherlone , and R. Darvizeh , “Comprehensive Comparison of Protective Effect of Customized Multi‐Layered Versus Boil‐And‐Bite Mouth Guard on Orofacial Tissues: A Finite Element Analysis Framework,” Proceedings of the Institution of Mechanical Engineers, Part C: Journal of Mechanical Engineering Science 237, no. 24 (2023): 5819–5828, 10.1177/09544062231167745.

[55] G. D. Lopes , J. D. Matos , D. A. Queiroz , et al., “Influence of Abutment Design on Biomechanical Behavior to Support a Screw‐Retained 3‐Unit Fixed Partial Denture,” Materials (Basel) 15, no. 18 (2022): 6235. 36143553 10.3390/ma15186235 PMC9504379

[56] A. M. Dal Piva , J. P. Tribst , A. L. Borges , C. J. Kleverlaan , and A. J. Feilzer , “The Ability of Mouthguards to Protect Veneered Teeth: A 3D Finite Element Analysis,” Dental Traumatology 1 (2022): 8. 10.1111/edt.12812 36573913

